# Human CD3γ, but not CD3δ, haploinsufficiency differentially impairs γδ versus αβ surface TCR expression

**DOI:** 10.1186/1471-2172-14-3

**Published:** 2013-01-21

**Authors:** Miguel Muñoz-Ruiz, Verónica Pérez-Flores, Beatriz Garcillán, Alberto C Guardo, Marina S Mazariegos, Hidetoshi Takada, Luis M Allende, Sara S Kilic, Ozden Sanal, Chaim M Roifman, Eduardo López-Granados, María J Recio, Eduardo Martínez-Naves, Edgar Fernández-Malavé, José R Regueiro

**Affiliations:** 1 Inmunología, Facultad de Medicina, Universidad Complutense, Madrid, 28040, Spain; 2 12 de Octubre Institute of Health Research, Madrid, Spain; 3 Departament of Pediatrics, Kyushu University, Higashi-ku, Fukuoka, Japan; 4 Inmunología, Hospital 12 de Octubre, Madrid, 28041, Spain; 5 Pediatric Immunology, Uludag University, Görükle-Bursa, Turkey; 6 Immunology Division, Hacettepe University, Children’s Hospital, Ankara, Turkey; 7 Immunology and Allergy, Hospital for Sick Children, University of Toronto, Toronto, Canada; 8 La Paz University Hospital, Madrid, Spain

**Keywords:** T cells, CD3, Haploinsufficiency

## Abstract

**Background:**

The T cell antigen receptors (TCR) of αβ and γδ T lymphocytes are believed to assemble in a similar fashion in humans. Firstly, αβ or γδ TCR chains incorporate a CD3δε dimer, then a CD3γε dimer and finally a ζζ homodimer, resulting in TCR complexes with the same CD3 dimer stoichiometry. Partial reduction in the expression of the highly homologous CD3γ and CD3δ proteins would thus be expected to have a similar impact in the assembly and surface expression of both TCR isotypes. To test this hypothesis, we compared the surface TCR expression of primary αβ and γδ T cells from healthy donors carrying a single null or leaky mutation in *CD3G* (γ^+/−^) or *CD3D* (δ^+/−^, δ^+/leaky^) with that of normal controls.

**Results:**

Although the partial reduction in the intracellular availability of CD3γ or CD3δ proteins was comparable as a consequence of the mutations, surface TCR expression measured with anti-CD3ε antibodies was significantly more decreased in γδ than in αβ T lymphocytes in CD3γ^+/−^ individuals, whereas CD3δ^+/−^ and CD3δ^+/leaky^ donors showed a similar decrease of surface TCR in both T cell lineages. Therefore, surface γδ TCR expression was more dependent on available CD3γ than surface αβ TCR expression.

**Conclusions:**

The results support the existence of differential structural constraints in the two human TCR isotypes regarding the incorporation of CD3γε and CD3δε dimers, as revealed by their discordant surface expression behaviour when confronted with reduced amounts of CD3γ, but not of the homologous CD3δ chain. A modified version of the prevailing TCR assembly model is proposed to accommodate these new data.

## Background

The human T cell antigen receptors (TCR) of αβ and γδ T lymphocytes are believed to assemble in a similar fashion [1]. First, variable αβ or γδ heterodimers bind to invariant CD3δε heterodimers, then to CD3γε heterodimers and finally to CD247 (or TCRζ) homodimers, resulting in surface TCR complexes with equal amounts of the two different, albeit highly homologous, CD3 heterodimers. In contrast, mouse αβ and γδ TCR differ drastically in their stoichiometry, since the γδ TCR does not incorporate any CD3δε dimers but, rather, two CD3γε dimers [2]. This finding begs the question as to whether the human variable αβ and γδ chains show identical affinity for both CD3 heterodimers.

We reasoned that, if both the αβ and the γδ TCR isotypes use identical amounts of CD3γε and CD3δε, then decreased availability of CD3γ or CD3δ proteins, as observed in heterozygous carriers of mutations in *CD3* genes [3], would be expected to have a similar impact on the assembly and surface expression of both αβ and γδ TCR isotypes. To test this hypothesis, we compared TCR surface levels of primary αβ and γδ T cells from healthy haploinsufficient donors carrying null or leaky mutations in *CD3G* (γ^+/−^) or *CD3D* (δ^+/−^, δ^+/leaky^). The results did not support the hypothesis of a similar impact on both TCR isotypes, but rather suggested a differential CD3γε and CD3δε usage scheme for each TCR isotype.

## Results

### Reduced surface αβ and γδ TCR expression in CD3γ^+/−^, CD3δ^+/−^ or CD3δ^+/leaky^ human T lymphocytes

**Figure 1:**
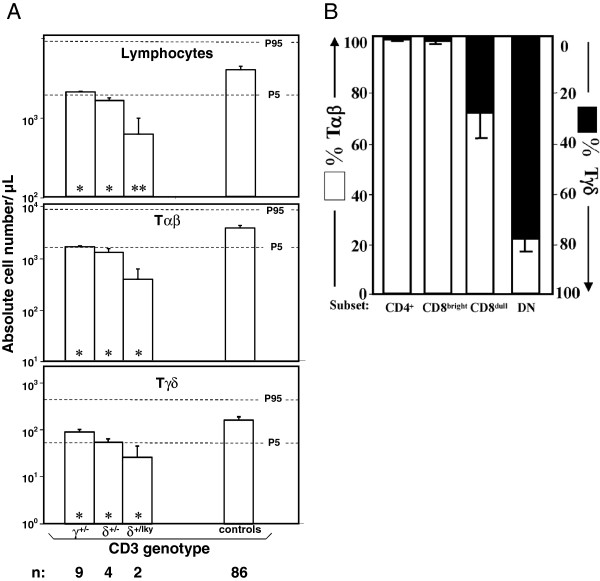
**Peripheral αβ and γδ T lymphocyte numbers in human *CD3G *or *CD3D *haploinsufficiencies. (A)** Peripheral blood cell counts from different γ^+/−^, δ^+/−^ or δ^+/leaky^ individuals are compared with the normal age-matched distribution as mean ± SEM against the P5/P95 normal range (horizontal dashed lines [6]). Asterisks in bars indicate significant differences as compared with controls. p < *0.05, **0.01 or ***0.001. **(B)** Proportion of αβ (BMA031^+^) and γδ (Immu510^+^) T cells (defined as CD3^+^) in different peripheral blood subsets (CD4^+^, CD8^bright^, CD8^dull^ and DN) in healthy individuals. Data are mean ± SD (n = 6).

*CD3G* (γ^+/−^) or *CD3D* (δ^+/−^) haploinsufficient donors were uniformly healthy and showed abundant peripheral blood T lymphocytes with an essentially normal phenotype (Table 1). However, total T cell numbers were consistently lower than controls (Figure 1A) which correlated with a partial impairment of lymphocyte function (Table 1).

**Table 1:** **Lymphocyte studies in haploinsufficient individuals**^**a**^

**CD3 GENOTYPE**	**γ**^**+/−**^	**δ**^**+/−**^	**δ**^**+/leaky**^	**Normal adults**
Number of subjects	4	2	2	12
Ages	46 ± 10	44	33 ± 1	37 ± 12
LYMPHOCYTE IMMUNOPHENOTYPE (%)				**Mean (range)**
T (CD3^+^)	60 ± 6	66 ± 1	62 ± 5	71 (54–77)
T (CD3^+^CD4^+^)	45 ± 4	39 ± 5	26 ± 1	43 (30–53)
T (CD3^+^CD8^+^)	18 ± 1	26 ± 6	16 ± 7	32 (16–39)
B (CD19^+^)	18 ± 5	12 ± 2	ND	12 (6–19)
NK (CD3^−^CD16^+^/CD56^+^)	17 ± 3	17 ± 2	9 ± 6	15 (8–31)
LYMPHOCYTE FUNCTION				
**T cell proliferation** (% of control max)^b^				**Normal control**
Medium	3 ± 1	1	8 ± 2	4 ± 3
Anti-CD3 (UCHT-1)	74 ± 4	ND	84 ± 6	100
Phytohemagglutinin (PHA)	61 ± 5	60 ± 4	**ND**	100
**Serum Ig (mg/dl)**		**ND**	**ND**	**Mean (range)**
IgG	790 ± 319			1158 (644–1436)
IgA	306 ± 54			200 (65–348)
IgM	47 ± 29			99 (55–206)
IgG1	611 ± 37			840 (380–1000)
IgG2	165 ± 222			240 (90–500)
IgG3	30 ± 6			80 (15–150)
IgG4	9 ± 6			40 (3–210)
**NK cell cytotoxicity (% lysis)**	52 ± 6	80 ± 5	**ND**	31 (21–41)

^a^Mean ± SD of the indicated number of different subjects. When available, multiple values from single donors were included as single means in the calculations. Data obtained from several sources, including published material [4,5].

^b^Percentage proliferation (H^3^-thymidine uptake for γ^+/−^ and δ^+/−^donors, CFSE dilution for δ^+/leaky^ donors) referred to the maximum response of a healthy control in each experiment.

**Figure 2:**
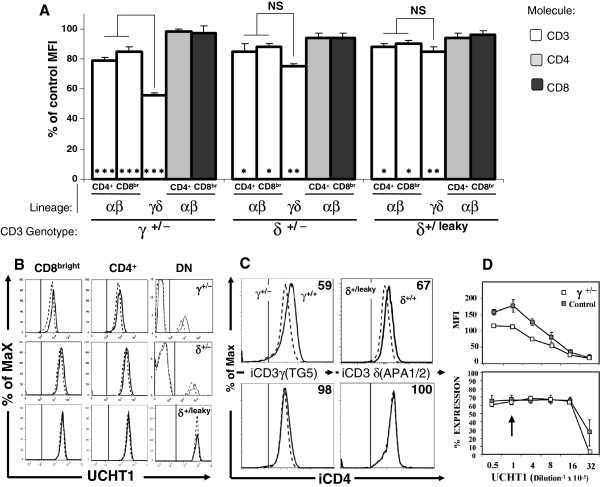
**Reduced surface αβ and γδ TCR expression in CD3γ**^**+/−**^**, CD3δ**^**+/− **^**and CD3δ**^**+/leaky **^**individuals using UCHT-1. **αβ T cells were defined as CD8^bright^ and CD4^+^ in 9 γ ^+/−^, 4 δ^+/−^, 2 δ^+/leaky^ and 7 ^+/+^ donors. Similar results were obtained with other anti-CD3 mAb (SK7, S4.1, F101.01, data not shown). (**A**) CD3 MFI ratios (X100) ± SEM relative to controls are shown for the indicated T cell lineages and genotypes. Asterisks in bars indicate significant differences as compared with controls, other comparisons as indicated. p as in Figure 1A. (**B**) Representative peripheral blood CD3 reactivity patterns of γ^+/−^, δ^+/−^ or δ^+/leaky^ T cells (dashed lines) as compared with ^+/+^ controls (solid lines). The vertical line in each panel indicates the upper limit of background fluorescence using isotype-matched irrelevant mAb. (**C**) Intracellular stainings of γ^+/−^ and δ^+/leaky^ T cells (dashed lines) as compared with ^+/+^ controls (solid lines). Vertical lines as in Figure 2B. The numbers in each histogram indicate MFI ratios (x100) relative to controls. (**D**) Comparative titration of CD3 (UCHT1) binding (MFI) to γ^+/−^ peripheral blood T cells (n = 1) *versus* +/+ controls (n = 2, mean ± SD). The percentage of bound cells was determined in parallel to establish the endpoint dilution (1:16.000). The arrow indicates the working dilution in all other experiments. Similar results were obtained using other antibodies (F101.01 or BMA031).

We have previously observed in γ^−/−^ individuals that CD3 expression levels are overestimated when T cells are defined using antibodies against TCR-associated epitopes [7], such as BMA031 (for TCRαβ) or Immu510 (for TCRγδ). To avoid a similar bias in haploinsufficient donors, TCR-independent electronic gates were first defined in order to identify αβ or γδ T cell subsets (Figure 1B). The results indicated that CD3^+^ cells within CD4^+^ or CD8^bright^ lymphocytes were >98% αβ T cells, whereas CD3^+^ double negative (DN) lymphocytes were 78 ± 6% γδ T cells. Accordingly, αβ and γδ T cells were gated as CD4^+^/CD8^bright^ and DN cells, respectively, for further analyses. Using several CD3-specific antibodies, analysis of surface TCR expression consistently showed reduced antibody binding in γ^+/−^ and δ^+/−^ T lymphocytes as compared to normal controls (50-90% as judged by their relative mean fluorescence intensity, Figure 2A, B). These results were confirmed in family members of two newly reported patients with a leaky mutation in *CD3D* (termed δ^+/leaky^) [8]. Consistent with their relatively higher CD3δ content as compared to δ^+/−^ donors, δ^+/leaky^ donors showed a milder, but nevertheless clear decrease in surface TCR expression (Figure 2A, B). In order to establish if these results were associated with reduced availability of each CD3 chain, we measured intracellular CD3γ (iCD3γ) or CD3δ (iCD3δ) by flow cytometry in haploinsufficient γ^+/−^ and δ^+/leaky^ donors. The results showed that this was indeed the case (Figure 2C), confirming previous reports of decreased CD3γ protein in haploinsufficient donors [3].

Serial dilutions of CD3 mAb further confirmed the findings above (Figure 2D), since the reduced binding to γ^+/−^ T cells persisted in saturation conditions, but it was gradually lost near the endpoint, supporting the existence of less CD3 binding sites [9].

From these results we conclude that human *CD3G* or *D* haploinsufficient donors show reduced binding of CD3-specific mAb to the TCR of their γδ and αβ T cells.

### Discordant reduction of surface αβ and γδ TCR expression in CD3γ^+/−^ but not CD3δ^+/−^ or CD3δ^+/leaky^ human T lymphocytes

**Figure 3:**
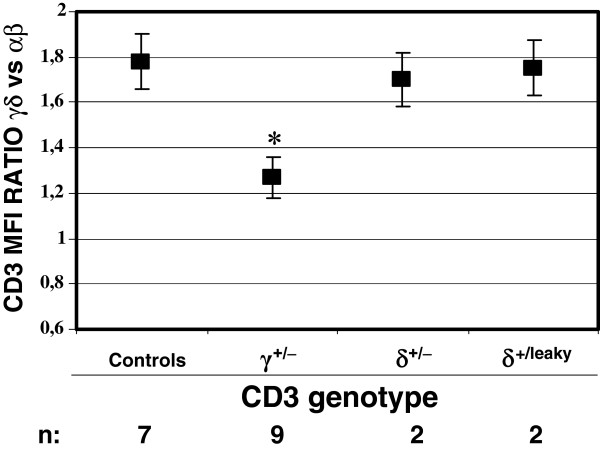
**Discordant reduction of surface αβ and γδ TCR expression in CD3γ**^**+/− **^**vs CD3δ**^**+/− **^**or CD3δ**^**+/leaky **^**individuals. **CD3 MFI ratios ± SEM of γδ *versus* αβ T cells for the indicated genotypes using UCHT-1. * indicates p < 0.05 as compared with other donors.

Analysis of CD3 mAb surface binding to αβ and γδ T cells with the different *CD3G* and *CD3D* genotypes, relative to normal controls, revealed that binding of CD3 mAb to γ^+/−^ γδ T cells was unexpectedly poor (55 ± 3%) as compared with γ^+/−^ αβ T cells (82 ± 8%, Figure 2A). This discordant reduction was specific for γ^+/−^ donors, as it was not observed in δ^+/−^ or δ^+/leaky^ donors. Further support for this discordant reduction was provided by the γδ *versus* αβ CD3 expression ratio, which is normally 1.9 ± 0.22 [10,11] but becomes significantly less in γ^+/−^ donors only (Figure 3). Taken together, these results indicate that normal surface γδ TCR expression in humans is more critically dependent on the relative availability of CD3γ, but not CD3δ, than that of the αβ TCR.

## Discussion

Human and mouse TCR complexes are assembled into octamers following common cues provided by transmembrane ionizable aminoacids in each dimer, with CD3γε and CD3δε ectodomains contributing additional extracellular interactions for their ordered incorporation into the complex [12]. The extracellular interactions are quite specific, as the mouse γδ TCR does not incorporate otherwise available CD3δε dimers, but rather two copies of the highly homologous CD3γε dimer. In sharp contrast, the human γδ TCR incorporates both [2]. Mammalian CD3γ, but not CD3δ, has a uniquely kinked ectodomain which fits into an asymmetrical loop in TCRβ for optimal αβ TCR assembly and expression, and cannot be easily replaced by CD3δ due to steric hindrance, with functional consequences [13]. This likely leads to the conserved structural asymmetry shared by the human and mouse αβ TCR. In contrast, TCRγ lacks the asymmetrical loop of its TCRβ homologue and seems to allow a less stringent (i.e., with less affinity) CD3 dimer usage in the γδ TCR, which may explain its disparate stoichiometry in the two species.

**Figure 4:**
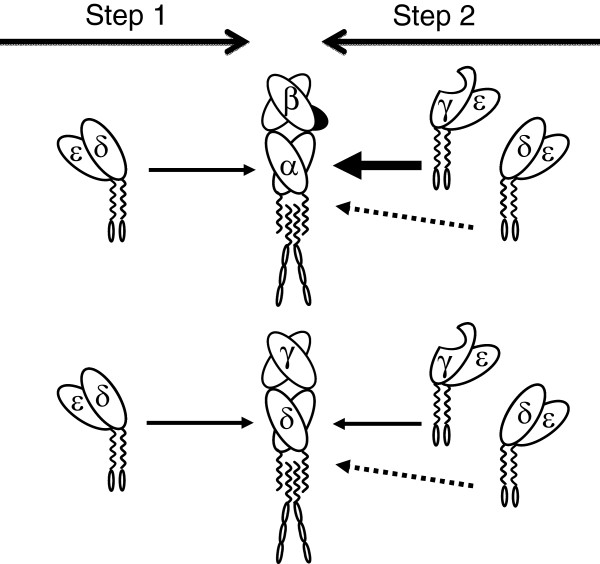
**Simplified model for the effect of human CD3 haploinsufficiencies on TCR assembly and expression. **Lack of a CD3γε-fitting structure in TCRγ, as opposed to TCRβ [13] (shown in black), and the resultant lower affinity of the former relative to the latter for the CD3γε dimer (represented by arrows), may explain the stronger impact of decreasing CD3γ (shown with a nick) but not CD3δ availability on surface γδ TCR expression as compared to that of the αβ TCR.

The present study suggests that there must be differential structural constraints for the building and stable expression of αβ and γδ TCR complexes, as revealed by their discordant behaviour in cell surface expression when confronted with reduced availability of CD3γ, but not of CD3δ (Figures 2 and 3). Our findings are in agreement with available information about the assembly of human surface αβ and γδ TCR [12], as explained above and as proposed in Figure 4. In the model, CD3δε dimers show a similar affinity for the human TCRα and TCRδ chains (step 1), thus reduced CD3δ expression has a similar impact on both. In contrast, when CD3γ is limiting, lack of a CD3γε-fitting structure in TCRγ, as opposed to TCRβ [13], may result in a lower affinity of the former relative to the latter for CD3γε. This may favour the incorporation of competing CD3δε dimers to nascent γδ TCR complexes (step 2), and would ultimately lead to γδ TCR receptors devoid of the required stability for optimal surface expression. Moreover, human TCRδ (but not TCRα) can stably recruit not only CD3δε but also CD3γε [14] during step1, which may reduce further the availability of CD3γε dimers for step2 when CD3γ is limiting (not shown).

Further studies are required to demonstrate a direct link between CD3γ or δ availability and TCR assembly and surface expression. However, the paucity of CD3 haploinsufficient individuals might hamper these studies in humans. A flow cytometry-based approach as illustrated in Figure 2B could help to identify such individuals.

Lastly, since carriers of *CD3G* or *CD3D* mutations showed affected TCR expression (Figure 2) and T-cell selection (Figure 1), which seemed in turn to impair to some extent T-cell function (Table 1) [15] the question of whether they also have increased risk of immunological dysfunction deserves further investigation.

## Conclusions

The results indicate that the dimer assembly scheme of the human TCR complex is different in αβ *versus* γδ T cells, as revealed by their discordant behaviour when confronted with limiting amounts of CD3γ, but not of the homologous CD3δ chain. The novel data allow proposing a modified version of the prevailing TCR assembly model.

## Methods

### Cells

After obtaining informed consent following IRB authorization (Ethics Committee for Clinical Investigation of Clínico San Carlos Hospital, Madrid), we studied nine healthy heterozygous carriers of mutations in CD3γ (γ^+/−^) [3,7] of Spanish or Turkish descent and six healthy heterozygous carriers of mutations in CD3δ (δ^+/−^, δ^+/leaky^) [8,16,17] of Japanese, Mennonite or Colombian descent. Normal donors (termed^+/+^) were used as controls. Their lymphocyte immunophenotype and functional features are summarized in Table 1. PBL were isolated by Ficoll-Hypaque (GE Healthcare) gradients and resuspended in RPMI 1640 medium (Gibco) supplemented with 10% FCS (PAA Laboratories), 1% L-glutamine and antibiotics-antymitotic (100 units/ml of penicillin G, 100 μg/ml of streptomycin sulfate, and 0.25 μg/ml of amphotericin B) from Gibco.

### Antibodies and flow cytometry

The expression of different surface markers was studied by flow cytometry using standard procedures in fresh whole blood or isolated PBL [18]. For intracellular stainings cells were fixed and permeabilized as previously reported [19].

The following anti-human mAb were used: anti-CD3ε (S4.1) from Caltag Laboratories (now Invitrogen), anti-CD3εγ/εδ (UCHT-1), anti-TCR αβ (BMA031), and anti-TCRγδ (Immu510) from Beckman Coulter, anti-CD3ε (SK7), anti-CD4 (Leu2a), anti-CD8 (Leu3a), anti-TCRγδ (11F2), and anti-CD8 (SK1) from BD Biosciences. Anti-CD3εγ/εδ (F101.01) hybridoma supernatant and anti-CD3δ (APA1/2) ascitic fluid were a generous gift from Dr. B. Rubin (CHU de Purpan, France). TG5 (an anti-CD3γ rabbit antiserum raised against the CD3γ C-terminal peptide GLQGNQLRRN) was kindly provided by D. Alexander (Babraham Institute, U.K.). The mAb were FITC-, PE-Cy5 or PE-conjugated, or purified, and for the latter a PE-conjugated goat anti-mouse IgG (H + L) or anti-rabbit (H + L) from Caltag Laboratories was used as a secondary reagent. Background fluorescence was defined with an isotype-matched irrelevant mAb from Caltag Laboratories. For comparative stainings we used the mean fluorescence intensity (MFI), defined as the average fluorescence value of the corresponding mAb referred to the logarithmic scale of fluorescence intensity along the x-axis of the histograms. Data were analyzed with FlowJo software (TreeStar).

### Proliferative assays

1×10^5^ PBLs were placed in round-bottom microtitre wells and stimulated with 10 μg/ml anti-human CD3 (UCHT-1) or 10 μg/ml PHA. After 3 days of *in vitro* culture, wells were individually pulsed with 1 μCi of ^3^H-TdR (Amersham, Buckinghamshire, U.K.) for another 16 to 18 h and harvested onto glass fiber filters. Thymidine incorporation into cellular DNA was evaluated as cpm in a scintillation βcounter (Packard, Meriden, CT).

For CFSE (carboxyfluorescein diacetate succinimidyl ester) dilution experiments, cells were labeled with 1 μM CFSE in PBS for 10 min at 37°C at day 0, washed twice in cold PBS, plated and stimulated as above. CFSE dilution was subsequently determined by flow cytometry within CD3^+^ lymphocytes.

### Cytotoxicity assays

Cytotoxicity was measured using the nonradioactive Cytotoxicity Detection kit LDH (Roche). Cells were cocultured in a 96 V-well plates for 4 h at 25: 1 (Effector: Target) ratios and the percentage of specific lysis was determined from the amount of lactate dehydrogenase activity detected in culture supernatants.

### Statistical analysis

Student’s *t*-tests were performed using SPSS 11.5.1 statistical program software (Chicago, IL). Only p values below 0.05 were considered significant. Data are presented as mean ± SEM (standard error of the mean) or ± SD (standard deviation).

## Abbreviations

PBL: Peripheral blood lymphocytes; MFI: Mean flourescence intensity; TCR: T cell antigen receptor; FCS: Fetal calf serum; ND: Not done; NS: Not significant.

## Competing interest

The authors declare no conflict of interest.

## Authors’ contributions

VP-F, ACG, BG, MM-R and HT carried out the analysis of TCR expression and function in human T lymphocytes MM-R, BG and MM-L performed titration studies and drafted the manuscript. LMA, SSK, OS, EL-G and CMF provided lymphocytes samples and leucocyte counts. MJR, EM-N, and EF-M provided technical knowledge and supervision. JRR planned the study and provided funds. JRR and EF-M wrote the manuscript. All authors read and approved the final manuscript.

## Authors’ information

E. Fernández-Malavé and J.R. Regueiro are joint senior authors.
